# Dr. Ira L. Jones

**Published:** 1913-10

**Authors:** 


Dr. Ira L. Jones, University of Buffalo, 1864, died at his home in Minetto, Oswego Co., N. Y., July 30, aged 81.

